# A Highly Conserved, Small LTR Retrotransposon that Preferentially Targets Genes in Grass Genomes

**DOI:** 10.1371/journal.pone.0032010

**Published:** 2012-02-16

**Authors:** Dongying Gao, Jinfeng Chen, Mingsheng Chen, Blake C. Meyers, Scott Jackson

**Affiliations:** 1 Center for Applied Genetic Technologies and Institute for Plant Breeding Genetics and Genomics, University of Georgia, Athens, Georgia, United States of America; 2 State Key Laboratory of Plant Genomics, Institute of Genetics and Developmental Biology, Chinese Academy of Sciences, Beijing, China; 3 Department of Plant and Soil Sciences, and Delaware Biotechnology Institute, University of Delaware, Newark, Delaware, United States of America; Louisiana State University, United States of America

## Abstract

LTR retrotransposons are often the most abundant components of plant genomes and can impact gene and genome evolution. Most reported LTR retrotransposons are large elements (>4 kb) and are most often found in heterochromatic (gene poor) regions. We report the smallest LTR retrotransposon found to date, only 292 bp. The element is found in rice, maize, sorghum and other grass genomes, which indicates that it was present in the ancestor of grass species, at least 50–80 MYA. Estimated insertion times, comparisons between sequenced rice lines, and mRNA data indicate that this element may still be active in some genomes. Unlike other LTR retrotransposons, the **sma**ll LTR **r**e**t**rotransposons (SMARTs) are distributed throughout the genomes and are often located within or near genes with insertion patterns similar to MITEs (miniature inverted repeat transposable elements). Our data suggests that insertions of SMARTs into or near genes can, in a few instances, alter both gene structures and gene expression. Further evidence for a role in regulating gene expression, SMART-specific small RNAs (sRNAs) were identified that may be involved in gene regulation. Thus, SMARTs may have played an important role in genome evolution and genic innovation and may provide a valuable tool for gene tagging systems in grass.

## Introduction

Transposable elements (TEs) are mobile DNA sequences found in most eukaryote genomes. Once considered “junk DNA”, transposons are now known to impact both gene and genome evolution [1]–[3]. In addition to their use for insertional mutagenesis, TEs are involved in many chromosome rearrangements, gene regulation and provide raw material for genetic innovation [4]–[6]. Furthermore, transposons also serve as essential components of heterochromatin maintaining centromeric and telomeric stability and heterochromatic silencing [7]–[9]. Transposons are divided into two major classes: Class II transposons that move to new locations via a ‘cut and paste’ model or by a rolling-circle mechanism; and Class I transposons or retrotransposons that mobilize through a ‘copy and paste’ model by which retrotransposon copies are integrated into new positions in the genome [10].

Long terminal repeat (LTR) retrotransposons are the most abundant mobile elements in the plant kingdom. In some plants, LTR retrotransposons can make up more than 70% of the genome [11]. The most typical features of LTR retrotransposons are direct LTRs that surround the internal domains (functional retrotransposases and/or other sequences) and are flanked by 4–6 bp target site duplications (TSDs). LTR retrotransposons are further subdivided into Ty1-copia (*Pseudoviridae*) and Ty3-gypsy (*Metaviridae*) superfamilies according to sequence divergence and the order of encoded gene products. Two other nonautonomous LTR-retrotransposons have been identified in plants, terminal-repeat retrotransposons in miniature (TRIM) and large retrotransposon derivatives (LARD) [12]–[15]. These two retrotransposons share similar sequence structures with Ty1-copia and Ty3-gypsy LTR retrotransposons but do not encode functional retrotransposases and their mobility is most likely catalyzed by other retrotransposons [16].

In contrast to LTR retroelements in other organisms, LTR retrotransposons in plants are often present in very high copy numbers. For instance, a single Ty1-copia retrotransposon family, BARE1, exists in the barley genome in more than 2×10^5^ copies and comprises about 9.6% of the genome [17]. Moreover, different LTR retrotransposons in plants can show distinct chromosomal distribution patterns. Some LTR retrotransposons are found in intergenic regions^1^ but most appear to be concentrated in highly heterochromatic regions (centromeres, pericentromeres, telomeres) [16], [18]–[23]. Furthermore, plant LTR retrotransposons are often large ranging from 4–10 kb, on average, and can even be as large as 18–22 kb and have LTRs that are over 5 kb [1], [24], [25]. Due to their replicative transposition and large sizes, the amplification of LTR retrotransposons can rapidly increase plant genome sizes over a relatively short time and is considered one of the primary contributors to the C-value paradox in plants [26]. For example, the genome size of a diploid wild rice, *O. australiensis*, is more than twice the diploid cultivated species and this is due to recent bursts of 3 LTR retrotransposon families which contribute more than 60% of the *O. australiensis* genome [27].

Active LTR retrotransposons not only can increase the host genome size but they can also result in deleterious mutations [1]–[3]. Thus, several strategies have evolved to prevent uncontrolled amplifications of LTR retrotransposons. First is the transcriptional silencing mechanism mediated through DNA methylation and chromatin modification to suppress transcriptional activity of transposons. Secondly, small RNA (sRNA) molecules can be incorporated into the RNA-induced silencing complex (RISC) and target LTR retrotransposons transcripts for post-transcriptional silencing [28], [29]. In addition, to counteract genome obesity, deletion of retrotransposons may occur through unequal homologous or illegitimate recombination between LTRs [21], [30], [31].

We discovered an unusually small, novel LTR retrotransposon named FRetro129 in *O. brachyantha*, a wild rice species, that is 292 bp with 85-bp direct terminal repeats. This is the smallest LTR retrotransposon reported thus far. Elements homologous to FRetro129 were found in other grass family genomes but not outside the grass family. Despite an ancient and/or possible multiple origins, FRetro129 and its homologues may yet be active in some genomes. Unlike most LTR retrotransposons in plants that are found in heterochromatic regions, this small retroelement is enriched within or near genes, a similar pattern to the DNA transposon, miniature inverted repeat transposable elements (MITEs). Our data indicates that the small retrotransposons may be involved in genic innovation and gene regulation. This small element family advances our knowledge about retrotransposons their role in gene/genome evolution and may provide a tool for functional gene studies in the grass family.

## Results

### Discovery of a new small retrotransposon in the *O. brachyantha* genome

In the process of annotating transposable elements (TEs) in the *O. brachyantha* genome, we identified a small element using the software LTR-Finder [32], which was only 292 bp including identical 85-bp terminal direct repeats (TDR) and flanked by 5-bp target site duplication (TSD). Database searches indicated no sequence similarity to any other described TEs. The element had a structure typical of LTR retrotransposon such as 5′TGT…ACA3′ terminal motifs, the presence of TDR and a 5-bp TSD. We named the novel element FRetro129. To our knowledge, this represents the smallest LTR retrotransposon reported so far. The internal sequence of FRetro129 was only 122 bp and did not encode any predicted protein, thus it is a non-autonomous element. Using FRetro129 as reference sequence to screen the *O. brachyantha* genome, 27 complete elements and 131 fragments were found. Even though the TDRs are very short (85 bp), eight solo LTRs were also found, which range in size from 79 to 87 bp and were flanked by 5-bp TSDs. The ratio of complete element to solo LTR was 3.4∶1. Sequence alignments between the reference element and other 26 complete elements indicated that some elements share less than 50% sequence identity with the 292-bp reference element, indicating that FRetro129 may be an ancient retrotransposon family based on accepted criteria [10]. However, we also found a full-length element with 99% sequence identity to the reference element indicative of recent amplification.

### Identification of homologous elements of FRetro129 in other genomes

**Figure 1:**
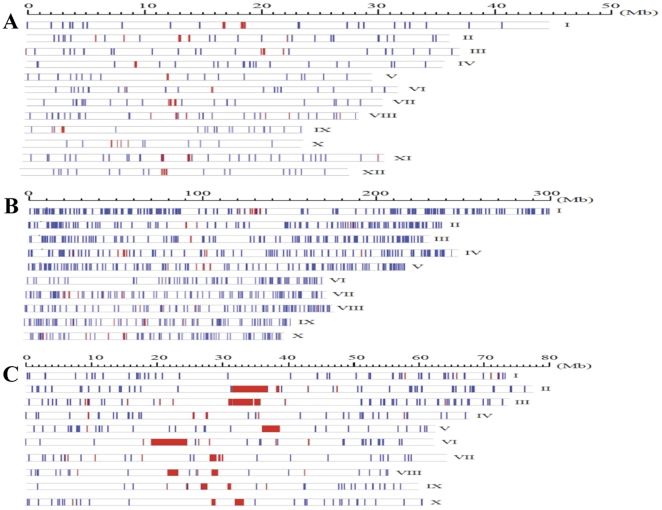
The distributions of FRetro129 homologs in Nipponbare (A), maize (B) and sorghum (C). The blue vertical lines mean the small elements and the red vertical bars indicate the centromere tandem repeats in rice (A), maize (B) and sorghum (C).

All 27 complete elements of FRetro129 family were used to identify sequences homologous to FRetro129 in other organisms. A total of 262 FRetro129 homologs were found in the Nipponbare (*Oryza sativa L.* ssp. *japonica*) genome, including 33 complete elements and seven solo LTRs (Table 1). The ratio of complete element to solo LTR is 4.7∶1, ∼1.4-fold higher than *O. brachyantha*. Unlike other LTR retrotransposons, such as CRR, *Dasheng* and FRetro3, which concentrate in and around centromeric regions [16], [20], [23], FRetro129 homologs were dispersed throughout the Nipponbare genome (Figure 1A). However, these elements were not evenly distributed across the 12 chromosomes, some chromosomes show higher transposon density than others. The average density of the small elements in the genome was 0.68 elements per Mb (total elements/sequenced rice genome size = 262/383 Mb). On chromosome 10, only eight FRetro129 homologous elements were identified resulting in an element density of 0.34 elements per Mb; in contrast, the density on chromosome 8 was nearly 3-fold higher, 1 element per Mb (28/28.5 Mb). The 93-11 (*Oryza sativa L.* ssp. *indica*) genome was also analyzed and 260 FRetro129 homologs, including 34 complete elements and five solo LTRs, were found (Table 1). It is interesting to note that two complete elements from Nipponbare and 93-11 share over 95% sequence identity with *O. brachyantha* FRetro129 elements.

**Table 1:** Distribution of FRetro129 in *O. brachyantha* and other 5 genomes.

Species	Copy number			
	Element	Solo-LTR	Fragment	Total
*O. brachyantha*	27 (260.6±35.5)	8 (84.0±2.4)	131	166
*O. sativa* (*Japonica*)	33 (283.7±17.8)	7 (83.2±1.1)	222	262
*O. sativa* (*In* ***d*** *ica*)	34 (279.7±19.4)	5 (82.2±1.1)	221	260
*Brachypodium distachyon*	45 (273.3±24.2)	8 (82.4±3.0)	555	608
Sorghum	99 (276.4±14.0)	2 (77.5±10.6)	387	488
Maize	347 (284.0±14.4)	14 (85.05±2.5)	1481	1842

Note: Numbers in () mean the average sizes (bp) of complete elements and solo-LTRs of FRetro129.

Database searches against GenBank and BAC end sequences (BESs) of 11 *Oryza* species (http://www.omap.org) identified several homologs of FRetro129 in 11 *Oryza* species including complete elements and solo LTRs (Table 2). The amount of FRetro129 homologs varied among the species. For instance, 138 FRetro129 homologs were found in *O. ridleyi* BES sequences, whereas, only 38 were found in *O. coarctata* BESs. These results indicate that FRetro129 is present across the *Oryza* genus.

**Table 2:** FRetro129 homologs in BAC end sequences of 11 *Oryza* species.

Species	Genome type	SequenceSize (Mb)	Copy number			
			Element	Solo-LTR	Fragment	Total
*O. glaberrima*	AA	39.4		1	65	66
*O. nivara*	AA	70.6			72	72
*O. rufipogon*	AA	50.0			45	45
*O. punctata*	BB	48.6	1		48	49
*O. minuta*	BBCC	94.8	4		66	70
*O. officinalis*	CC	72.5			52	52
*O. alta*	CCDD	75.5	2	1	39	42
*O. australiensis*	EE	80.4	1		25	26
*O. granulata*	GG	93.2	2		74	76
*O. ridleyi*	HHJJ	129.4			138	138
*O. coarctata*	HHKK	129.0	1		37	38

We next screened whole genome sequences from maize, sorghum and *Brachypodium distachyon* (*Brachypodium*). A total of 488 and 608 homologous elements of FRetro129 were detected in sorghum and *Brachypodium*, respectively. More than 1800 FRetro129 homologs including 347 complete elements and 14 solo LTRs were found in the maize genome. The ratios of complete element to solo LTR are 50∶1, 24.7∶1 and 5.6∶1 for sorghum, maize and *Brachypodium*, respectively. The highest and lowest ratios of complete element to solo LTR are in sorghum and *O. brachyantha*, respectively (Table 1). FRetro129 homologous elements were distributed throughout the genomes of maize, sorghum (Figure 1B & C) and *Brachypodium* (data not shown).

The FRetro129 elements were used as queries to conduct BLASTN searches against GenBank, six and seven complete elements of FRetro129 were found from expressed sequence tags (ESTs) of sugarcane (*Saccharum*) and switchgrass (*Panicum virgatum*), respectively. One complete homologous element of FRetro129 was identified from foxtail bristlegrass (*Setaria italica*) genomic sequence. In addition, fragments of FRetro129 with significant similarity (E value<10^−5^) also were found in wheat (*Triticum aestivum*), barley (*Hordeum vulgare*), pearl millet (*Pennisetum glaucum*), perennial triticeae, meadow fescue (*Festuca pratensis*), perennial ryegrass, tall fescue (*Festuca arundinacea*), bluebunch wheatgrass (*Pseudoroegneria spicata*), Canada wild rye (*Elymus wawawaiensis*), wild oat (*Avena barbata*) and two bamboo species (*Sasa kurilensis* and *Phyllostachys edulis*). No significant sequences matches were found in genomes outside the grass family, which was determined using BLASTN searches against genome sequences from Arabidopsis, papaya, soybean, grape vine and poplar. This suggests that FRetro129 and its homologous elements are either restricted to the grass family, or absent or highly diverged in the other genomes.

**Figure 2:**
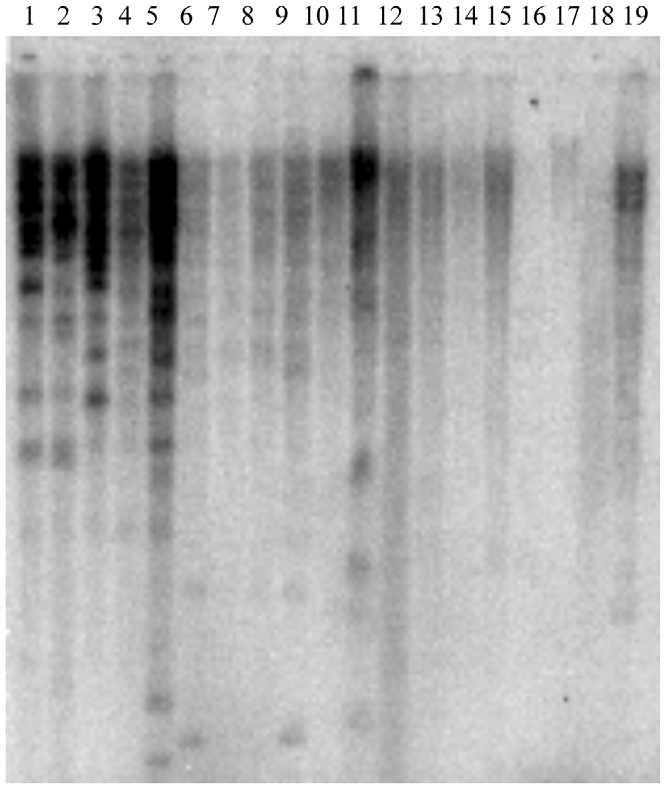
Southern blot of genomic DNA from 19 plants with FRetro129 probe. 1. Nipponbare; 2. *O. glaberrima*; 3. *O. nivar*a; 4. *O. rufipogon*; 5. *O. longistaminata*; 6. *O. punctata*; 7. O. officinalis; 8. *O. minuta*; 9. *O. australiensis*; 10. *O. ridleyi*; 11. *O. brachyantha*; 12. *O. granulate*; 13. Maize; 14. Wheat; 15. Barley; 16. Arabidopsis; 17. Tomato; 18. Soybean; 19. Sorghum. Genomic DNA was digested with *EcoR* I.

To further verify the presence of FRetro129 in grass species, DNAs from 19 plant species were digested with *EcoRI* and hybridized using FRetro129 as probe. The strongest signals were found in *O. brachyantha*, Nipponbare and four other AA *Oryza* species indicating the abundance of FRetro129 in these genomes. Hybridization signals were detected in other wild rice species, maize, wheat, barley and sorghum but the hybridization signals were not as strong as *O. brachyantha* and Nipponbare (Figure 2). This may be due to fewer copies of the small element but is most likely due to sequence divergence with the FRetro129 probe. For instance, based on sequence analysis there are more than 1800 small elements in maize but no signal was observed in the Southern blot. No hybridization signal was observed in Arabidopsis, soybean and tomato (Figure 2). Therefore, our Southern blot analysis confirmed that FRetro129 and it homologs are restricted to the grass species. We refer to FRetro129 and its homologs as **sma**ll LTR **r**etro**t**ransposons (SMARTs).

### Phylogenetic analysis of SMARTs

**Figure 3:**
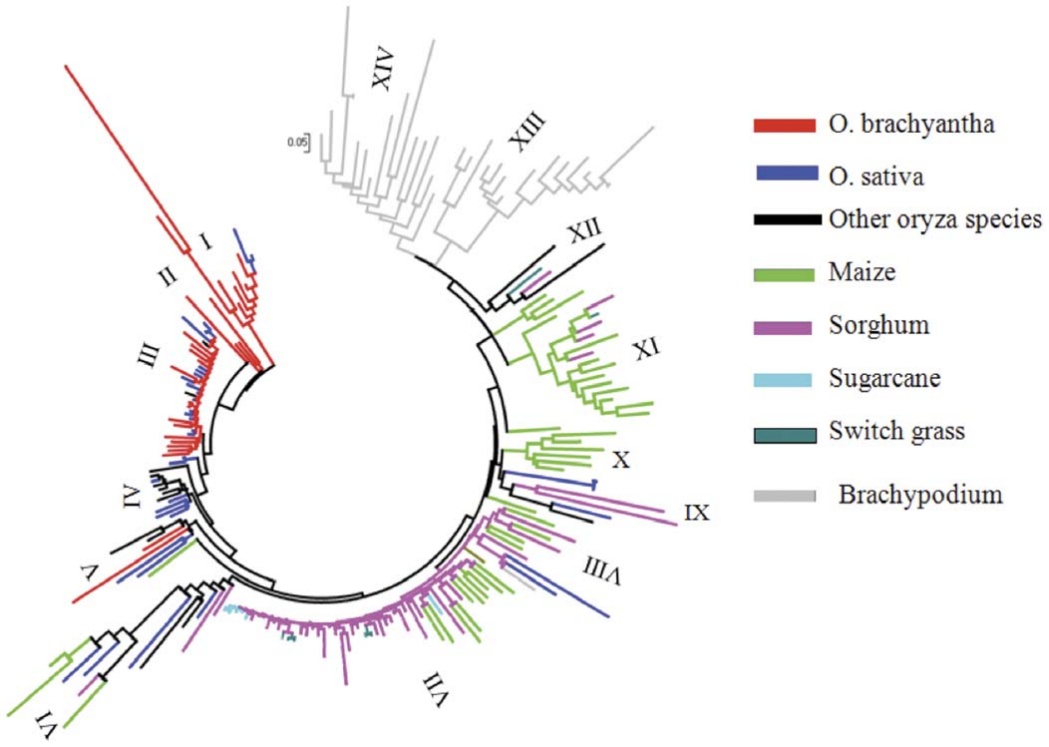
Phylogenetic tree of 200 complete elements from rice species and other grass genomes.

To provide more insight into the sequence diversity and evolutionary relationship of SMARTs from different species, 200 complete elements identified from 18 genomes were used to generate a phylogenetic tree. The results showed that the SMARTs clustered into 14 subfamilies (Figure 3). Elements from one genome can be grouped into multiple subfamilies. For instance, 27 elements in *O. brachyantha* fall into four subfamilies (I–IV; Figure 3). Some branches of the four subfamilies are very divergent, suggesting that the FRetro129 may be an ancient retrotransposon family. Only four subfamilies (II, X, XIII, XIV) have elements from a single species, the other 10 subfamilies contained transposon sequences from at least 2 species. For example, subfamily VI included 12 complete elements from rice, maize, sorghum and 2 wild rice species (*O. alta* and *O. granulata*), even though rice diverged from a common ancestor with sorghum and maize ∼50–80 MYA [33], [34]. Sequence alignments showed that the sequence identities of some complete elements from different genomes were higher than that from within the same species. For example, identity between FRetro129 and some copies in *O. brachyantha* is less than 60%, whereas it is over 80% identity with some elements from sorghum and maize (Figure S1). These results indicate the existence of multiple ancient lineages of SMARTs in the grass family that likely diverged before the radiation of rice and other genomes.

### The insertion time of SMARTs in *O. brachyantha* and other genomes

**Figure 4:**
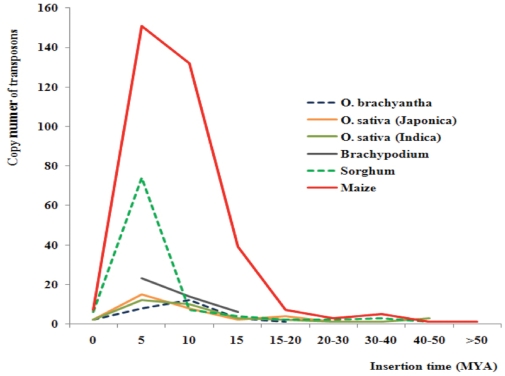
Insertion times of complete SMART elements from six grass genomes.

When an LTR retrotransposon is inserted into a genome, the two LTR sequences are identical at the time of insertion. Subsequently, both LTRs diverge due to independent accumulation of mutations. Thus, the insertion date of LTR retrotransposon can be estimated based on sequence divergence between LTR sequences [35]. Insertion times of all intact SMARTs from six species in the grass family for which whole genomes sequences were available were calculated using this approach (Figure 4).

Among 27 complete elements of FRetro129 in *O. brachyantha*, 12 elements (44%) integrated into the genome 5–10 MYA, five elements (18.5%) inserted into the genome more than 10 MYA, including an element that is estimated to have integrated into the genome about 36.9 MYA. These results suggest that FRetro129 is an ancient transposon family, consistent with phylogenic analysis of FRetro129 members (Figure 3). However, the insertion times of two elements were 0 MYA indicative of very recent insertions and suggests that FRetro129 may still be active or that the time since insertion was not longer enough for divergence of LTRs.

The insertion times of 33 intact SMARTs in Nipponbare range from 0 to 38.6 MYA, again indicating that this small retrotransposon is older than the genus. LTR sequences of two complete elements, located on chromosomes 9 and 11, were identical. It is interesting that we also found two intact elements in 93-11 that also integrated into the genome recently (0 MYA), however, they are located on chromosome 2 and 3 and have different TSDs from the two youngest Nipponbare elements. Thus, the small element has recently been active in Nipponbare and 93-11, since they diverged ∼0.2–0.4 MYA [36], [37].

The insertion times of complete elements from sorghum, maize and *Brachypodium* were also analyzed. Although several elements were found that inserted more than 30 MYA in all three genomes, the burst peaks were 0–5 MYA. Very recent insertions of 0 MYA were found in sorghum (six elements) and maize (seven elements) again supporting recent transposition of these elements in grass genomes.

Taken together, the data revealed that FRetro129 and its homologs represent an ancient family and that their amplification occurred over a long period and that they may still be active in some genomes.

### SMARTs preferentially insert into/near genes and can affect gene structure

The availability of a large collection of full-length cDNA sequences [38] and extensive rice genome annotation resources [39], [40] allowed us to determine the integration sites of the small elements relative to genes. A total of 262 SMARTs in Nipponbare including 33 complete copies and seven solo LTRs were examined. Of these sequences, 74 (28.2%) were in introns of annotated rice genes. Three and eight were located in exons and untranslated regions (UTRs), respectively. In addition, 53 (20.2%) were found within one kb upstream or downstream of annotated genes. 28.6% of the sequences were harbored in single-copy regions with no annotated genes. The remaining sequences were located in either transposons or multiple-copy regions (Table 3). Taken together, about 53% of the SMARTs in Nipponbare were located within or near genes. This suggests that SMARTs preferentially integrate, or are retained in genic regions, especially introns.

**Table 3:** Insertion sites of SMARTs in rice, sorghum and maize.

Location of small element	Rice	Sorghum	Maize
Gene	85 (7e+3s+75f)	64 (62e+2s)	205(200e+5s)
intron	74 (6e+3s+65f)	64 (62e+2s)	204(199e+5s)
exon	3(3f)		
UTR	8 (1e+7f)		1(1e)
Within 1 Kb flanking gene	53 (8e+1s+44f)	11(11e)	39(34e+5s)
Single copy, no annotated gene	75 (10e+1s+64f)	23(23e)	90(87e+3s)
Other	49 (8e+2s+39f)	3(3e)	25(24e+1s)
Total	262(33e+7s+222f)	101(99e+2s)	361(347e+14)

Note: e, s and f mean complete element, solo LTR and fragment, respectively.

We next investigated the location of full-length elements and solo LTRs in sorghum and maize. Of 99 complete elements in sorghum, 62 (62.6%) and 11 (11.1%) were found in introns or within 1 kb of a gene, respectively. Additionally, two solo LTRs were found in the 4^th^ intron of *SORBIDRAFT_10g031030* and the 5^th^ intron of a annotated gene supported by the maize cDNA sequence (GenBank accession: NM_0011579900). Four other genes, *SORBIDRAFT_03g004580*, *SORBIDRAFT_04g011760*, *SORBIDRAFT_06g024520* and *SORBIDRAFT_10g004493*, each contained two complete elements in different introns. In maize, of 361 SMARTs including 347 complete elements and 14 solo LTRs, 204 (56.5%) and 39 (10.8%) of the sequences were in introns or 1 kb of a gene, respectively (Table 3). These results support the observation in rice that SMARTs exhibit an insertion or retention preference to genic regions, especially introns.

We compared gene sequences with insertions of the SMARTs to orthologous or/and paralogous genes, and we analyzed 50 sorghum genes and 83 maize genes to determine if insertions of SMART elements affected gene structures or splicing sites. Of the 50 genes in sorghum, seven genes did not have expressed orthologous genes in either maize or rice and 36 genes had the same gene structure as their orthologous/paralogous genes. In maize, six of the 83 genes had no expressed counterpart in either sorghum or rice and 73 genes had identical structures as their orthologs/paralogs. Thus, 84% (36/(50-7)) of the genes in sorghum and 95% (73/(83-6)) of the genes in maize with SMART insertions did not result in altered gene structures. These results indicate that as a general rule SMART insertions do not affect the gene structures. However, for 11 genes [sorghum (7) and maize (4)] gene structures were altered relative to their orthologs and/or paralogs (Table S1).

**Figure 5:**
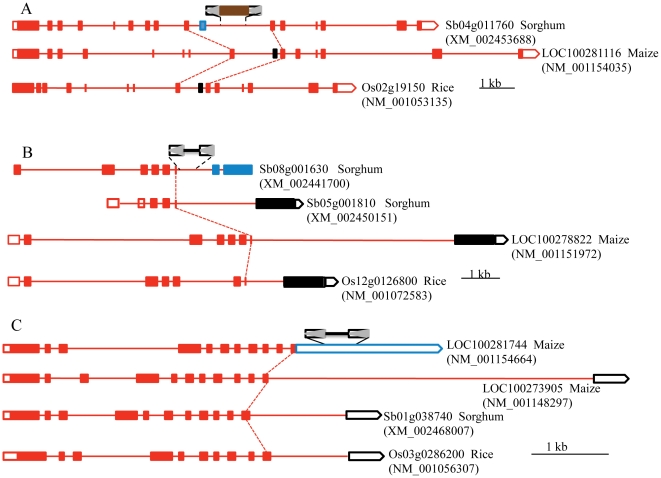
Changes in gene structures mediated by SMART insertions. A. Gene models of *Sb04g011760* in sorghum and its orthologous genes *LOC100281116* in maize and *Os02g19150* in rice. *Sb04g011760* gene contains a nested block of SMARTs. Brown rectangle represents the nested and truncated SMART. Blue rectangle represents the unique exon for *Sb04g011760* and the black rectangles are the exons present in orthologous genes from maize and rice. Red dashed lines indicate shared exons. B. Gene models of *Sb08g001630* and its paralogous gene *Sb05g001810* in sorghum and the orthologous genes *LOC100278822* in maize and *Os12g0126800* in rice. *Sb08g001630* contains a SMART. C. Gene models of maize gene, *LOC100281744*, and its paralog *LOC100273905* and orthologs *Sb01g038740* in sorghum and *Os03g0286200* in rice. Blue box represents the 3′ UTR of *LOC100281744* which contains a SMART and the black box represents 3′UTR of the paralogous and orthologous genes. The cDNA sequence for each gene model is shown in ().

Three exemplars are described where all the gene structures are supported by full-length cDNAs. The sorghum gene, *Sb04g011760*, harbors a nested block in which one small element contains another truncated copy. Compared to the orthologous genes from maize and rice, the 10^th^ exon of *Sb04g011760*, adjacent to the nested block, is unique for sorghum. *Sb04g011760* gene lacks a 75-bp exon that is present in the orthologous genes from maize and rice (Figure 5A). A small element was found between 6^th^ and 7^th^ exons of another sorghum gene, *Sb08g001630*. The first 6 exons of *Sb08g001630* are the same as the orthologous genes, however, the last 2 exons differ from the orthologous genes in maize and rice and the paralogous sorghum gene, *Sb05g001810* (Figure 5B). The structure of the 11 exons of the maize gene, *LOC100281744*, are identical to the orthologous genes and the paralogous gene. However, gene *LOC100281744* has a much longer 3′ UTR (1868 bp) that contains the small element sequence. The 3′ UTRs of the orthologous genes and the paralogous gene are separated by the intron and vary in size from 459 to 546 bp (Figure 5C).

### Recently inserted SMARTs affect gene transcription

**Figure 6:**
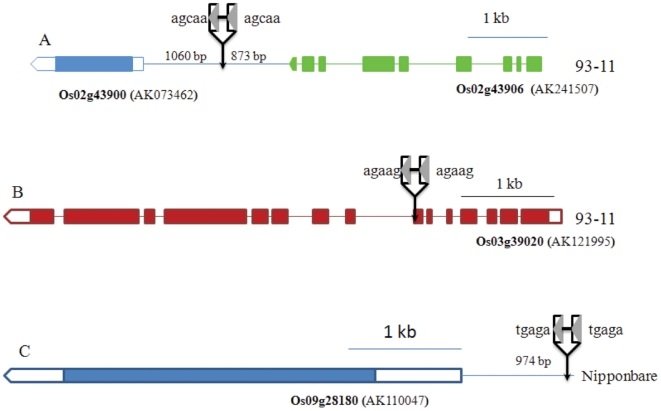
Three new SMART insertions located in or near rice genes. A. SMART element inserted in the internal region between 2 genes, *Os02g43900* and *Os02g43906*. The orthologous region in Nipponbare does not have the element. B. an element inserted in the seventh intron of *Os03g39020* in 93-11 and is absent in the orthologous gene in Nipponbare. C. an element located 974 bp upstream of *Os09g28180* in Nipponbare and absent in the orthologous gene in 93-11.

Through sequence comparisons of SMARTs and their flanking regions between 93-11 (*Indica*) and Nipponbare (*Japonica*), we found insertions that occurred after the split of two rice subspecies, ∼0.2–0.4 MYA [36], [37]. A new insertion is defined as the presence of a SMART element and 5-bp TSD in one species but not found in the orthologous region. Five new insertions were identified in 93-11, three of which were located in non genic regions and the other two located in the intergenic region of *Os02g43900* and *Os02g43906* and the seventh intron of *Os03g39020* (Figure 6 A–B). One new insertion was found in Nipponbare 974 bp upstream of *Os09g28180* (Figure 6 C). In addition, five complete elements from Nipponbare and their flanking 200 bp were not found in 93-11. However, given that the 93-11 genome is not completely sequenced, these five elements may not have been captured in the 93-11 genome assembly. To determine if these elements were present in 93-11, PCR was performed using flanking sequence primers for an element that inserted into the 3′ UTR of *Os09g25945*. No PCR product were found in 93-11 indicating that the flanking sequences and the element are either deleted or not present in 93-11. It was difficult to confirm the other four elements by PCR as they are located in retrotransposons. Thus, at least six new insertions were identified that occurred after the divergence of 93-11 and Nipponbare.

**Figure 7:**
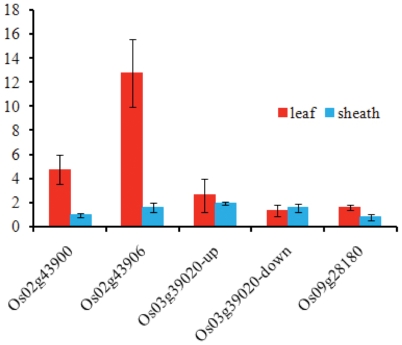
qRT-PCR results of 4 genes, *Os02g43900*, *Os02g43906*. *Os03g39020* and *Os09g28180*. Expression levels of the genes with SMART insertions relative to the orthologous genes without the transposon expressed as fold change (y axis). Error bars indicate the standard error of biological replicates.

Quantitative real time PCR (qRT-PCR) was used to determine transcription levels for four genes polymorphic for SMART insertions between Nipponbare and 93-11 (*Os02g43900*, *Os02g43906*, *Os03g39020* and *Os09g28180*; Figure 7). The fold change in gene expression levels were used to estimate the potential effect that the SMART element has on expression. For *Os02g43900*, no change of relative expression level was seen in the sheath, but the gene copy with the SMART element was expressed five-fold more in the leaf. The expression level of *Os02g43900* in leaf and sheath of 93-11 were approximately 13 and 2 times higher than that in Nipponbare. These results suggest that the intergenic insertion may have resulted in increased expression in leaf tissues for both *Os02g43900* and *Os02g43906*. For *Os03g39020*, we designed two pairs of primers upstream and downstream of the intronic insertion. The gene expression levels increased 1.3 to 2.6 times with the two sets of primers, thus this intronic insertion in 93-11 appears to have little or no effect on gene expression. For *Os09g28180*, which has an insertion about 1 kb upstream in Nipponbare, the gene expression is only slightly increased in leaf tissue and no change in expression level was found in the sheath.

### Small RNA (sRNA) target SMARTs in rice and maize

Small RNAs play essential roles in plant development, responses to various environmental stresses, and the class of heterochromatic siRNAs function predominantly in transposon silencing [29], [41]. In order to identify sRNA molecules that originate from and target SMARTs, the SMART elements from rice and maize were used as queries to perform BLASTN searches against the sRNA database from rice (http://mpss.udel.edu/rice_sbs) and maize (http://mpss.udel.edu/maize_WGS) and the Cereal Small RNA Database (CSRD, http://sundarlab.ucdavis.edu/smrna). After removing the redundant sequences, a total of 324 distinct sRNAs in rice and 77 sRNAs in maize were identified, which showed 100% sequence identity to the SMART elements. Of these, 40 randomly selected sRNAs (20 from each species) were used as queries to search against the rice and maize genomes to map their distributions. All matched sites for the 40 sRNAs mapped to the locations of SMARTs in rice and maize. Among these selected sRNAs, only four sRNAs in rice and three sRNAs in maize matched exactly one site (Table 4), while 16 of the 20 (80%) from rice and 17 of 20 (85%) from maize had exact matches to multiple genomic locations, with an average of 13.4 and 43.4 matched locations per small RNA in rice and maize. This degree of repetitiveness was consistent with our estimates for the total number of SMART elements in these genomes. Some sRNAs, for example zma-smRNA215152 and zma2-smRNA2034598 (from CSRD), had identical matches to more than 100 loci, suggesting that for some elements, there may be a larger number of more distant relatives. In addition, we noticed that some of the SMART-derived sRNAs are conserved across the grasses. For example, a 24-nt rice small RNA, osa-smRNA15336, exactly matched 63 SMARTs from rice, *O. alta*, maize and sorghum and had 1 bp mismatch with SMARTs from *O. brachyantha*, *O. minuta*, sugarcane and foxtail millet (Figure S2). Thus, the SMART elements are likely silenced in genomes of diverse species.

**Table 4:** Small RNA families in rice and maize matching the small LTR retrotransposons.

Familiy	Sequence (5′-3′)	Size (nt)	Total matched sites		
			Transposon	Other s	Total
osa-smRNA87	cgaguucgaauccuggcuggcgc	23	14	0	14
osa-smRNA1629	gggggucucgcgugaggggg	20	15	0	15
osa-smRNA11575	gcaugcaacucaauaugguaucag	24	4	0	4
osa-smRNA15336	cucgcgugagggggaguguuggag	24	15	0	15
osa-smRNA26529	ugcaccagccaguugcaccuaaa	23	23	0	23
osa-smRNA27409	ugagaagaccuugugugaggggga	24	1	0	1
osa-smRNA28265	uggugcaugcaacuuaauauggua	24	16	0	16
osa-smRNA41835	ggcuuuuaggugcaauuggcuggu	24	2	0	2
osa-smRNA55476	gaguguauaaagugaauugcccgc	24	26	0	26
osa-smRNA60213	agcuuaggcuuuuaggugcaugca	24	1	0	1
osa-smRNA73491	cagccaauuacaccuaaaagccu	23	2	0	2
osa-smRNA83486	cccccucaagucucaagcgugga	23	24	0	24
osa-smRNA95248	cucgcgugagggggaguguuggag	24	15	0	15
osac11smRNA288	ccuaagcugauagggaaagauggg	24	4	0	4
osa-smRNA96292	ucggugcaugcaacucaauaugg	23	1	0	1
osac1-smRNA38	cuaaaagccuaagcugauagggaa	24	24	0	24
osa-smRNA109754	aucaguuuaggcuuuuaggugcaa	24	3	0	3
osa-smRNA117590	uuaaucuuuuggguugaacug	21	2	0	2
osac1-smRNA25	aucagcuuaggcuuuuaggugcaa	24	23	0	23
osa-smRNA129401	aucagcuuaggcuuuuagguguaa	24	3	0	3
osa-smRNA131555	aggcuuuuagguguaacugacuga	24	1	0	1
zma-smRNA2592	gcaugcaccaaccaauucaaccca	24	45	0	45
zma-smRNA7727	acccaaaagcuuaagcugaugaga	24	8	0	8
zma-smRNA179230	acgagacucuuuuaggucccugac	24	1	0	1
zma1-smRNA578417	agcuuaagcugaugggaagaggu	23	11	0	11
zma-smRNA207113	ugaguugaacugguuaaugcgucc	24	1	0	1
zma-smRNA215152	auuaaauaaaauaauuguugc	21	106	0	106
zma1-smRNA 619577	uuggauugaauugguuggugc	21	6	0	6
zma-smRNA237200	uauaaguggauugucuacauucuc	24	1	0	1
zma1-smRNA 1214156	gcucgcuccuauauuccacgucag	24	15	0	15
zma1-smRNA 965412	aagcuuuuggguugaacugguugg	24	24	0	24
zma1-smRNA 902714	aaauaaaauaauuguugcucgcuc	24	80	0	80
zma1-smRNA 852339	auaaaauaauuguugcucgcuccu	24	55	0	55
zma2-smRNA 2034598	aucagcuuaagcuuuuggguugaa	24	123	0	123
zma2-smRNA 1395336	uugguuggugcaugcaacuuaaua	24	91	0	91
zma2-smRNA 1368217	agccagaggucucgaguucgaauc	24	49	0	49
zma2-smRNA 1228712	uccuauauuccacgucagagaccc	24	13	0	13
Zma3-smRNA 1484487	ugcucgcuccuauauuccacguca	24	16	0	16
Zma3-smRNA 948277	ucgcuccuauauuccacgucaga	24	21	0	21
Zma3-smRNA 874834	gaauccugguuagcacaauuaaau	24	23	0	23
Zma3-smRNA 580387	aguguuggaauauaauauaaguga	24	51	0	51

Note: 1) os-smRNAs and zma-smRNA represent small RNA families from rice and maize, respectively; 2) Target sites should share 100% sequence identity with the small RNAs.

**Figure 8:**
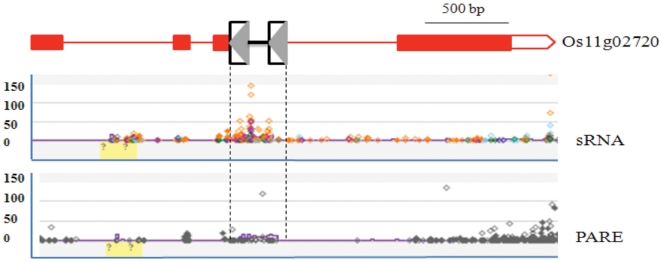
Comparison of the gene models and the sRNA and PARE expression patterns. *Os11g02720* contains an intronic element. The y axis indicates the adundance of sRNAs or PARE reads. Red boxes and lines indicate the exons and introns of the gene, respectively, and yellow shading represents sequences masked by the TIGR rice repeat database. The sRNA or PARE reads are shown as diamonds and the diamonds with different colors represent different sRNA size classes.

We also analyzed a set of strand-specific mRNA data derived from uncapped or cleaved mRNAs [42]. These “parallel analysis of RNA ends” (PARE) data are typically used to identify targets of microRNAs; PARE tags are derived from poly-A transcripts, and are thus indicative of normal gene expression. In order to explore the possibility that SMARTs may be actively expressed, with some elements escaping silencing by the sRNAs described above, 10 genes in which SMARTs were found were searched against the rice PARE database (http://mpss.udel.edu/rice_pare). A total of 80 PARE signatures were identified that exactly matched these SMARTs but not the flanking protein-coding genes (Figure 8, Table S2). These data suggest that at least some SMART elements are actively expressed.

### Identification of a candidate autonomous element

**Figure 9:**
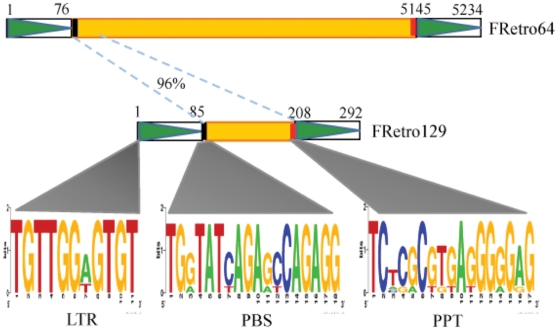
Structures of FRetro129 and FRetro64. Boxes with a green triangles and the yellow boxes represent LTRs and internal region of the LTR retrotransposons, respectively. Small black and red box show primer binding sites (PBS) and poly-purine tracts (PPT). Broken blue lines indicate the internal region shared between FRetro129 and FRetro64. The Black lines indicate the conserved 11-bp motif of LTR, PBS and PPT. Grey triangles are conserved motifs of LTR, PBS and PPT shared by FRetro129 and FRetro64.

Since FRetro129 elements have no coding capacity,transposition must depend on transposases encoded by other transposons. In order to identify potential autonomous transposable element(s) responsible for movement of SMARTs, FRetro129 elements were used as queries to search against the *O. brachyantha* genome sequence and the transposable element database of *O. brachyantha* (Gao et al., unpublished data). An LTR retrotransposon named FRetro64 showed sequence similarity with the FRetro129 element (Figure 9). FRetro64 is 5,234 bp including 76 and 90 bp LTR sequences. The internal sequence of FRetro64 was used as a query to conduct BLASTX searches and revealed that FRetro64 belongs to the *Ty1-copia* superfamily. The internal sequence of FRetro64 shares 96% identity with the internal sequence of FRetro129 (Figure 9). LTR sequences between FRetro129 and FRetro64 do not show detectable similarity using BLASTN2 program; however, they do share an 11-bp motif. FRetro129 and FRetro64 also share similar primer binding sites (PBS) and poly-purine tracts (PPT) sequences (Figure 9). FRetro64 is the only element in the *O. brachyantha* genome that has detectable sequence similarity with FRetro129. Thus, FRetro64 is the putative autonomous element that catalyzes FRetro129 transposition.

Some homologs of FRetro129 present in other genomes of the grass family appear to be recent transpositions, thus autonomous elements should be present in these species. Three strategies were used to detect possible autonomous elements in other genomes: 1) the small elements were used to screen their host genomes; 2) the small elements were used to search against the available transposon databases, including that from GIRI (http://www.girinst.org/), TIGR_Plant_Repeats (ftp://ftp.plantbiology.msu.edu/pub/data/TIGR_Plant_Repeats/). For rice, we also searched against RetrOryza (http://www.retroryza.org/) and the rice transposon library (Dr. Ning Jiang, Michigan State University, unpublished); and 3) FRetro64 was used as a query to conduct BLAST searches against GenBank. No putative retrotransposons were found when using the SMARTs to search against the genome sequences or transposon database which suggests that either the autonomous elements were missed by these transposon database or that the autonomous elements may not have any detectable similarity with their non-autonomous elements. However, several retrotransposons were identified from maize, sorghum, sugarcane and switchgrass using FRetro64 as a query. They range in size from 4871 to 5785 bp and had 105–117 bp LTRs, we named these elements ZM64 (FRetro64 homologous retroelement in maize), Sor64 (in sorghum), Sugar64 (in sugarcane) and Swit64 (in switchgrass). All four retrotransposons shared 70–78% sequence identity with the internal sequence of FRetro64 whereas their LTRs have no sequence similarity with LTRs of FRetro64. However, the four retroelements share sequence similarity with both LTRs and internal region between each other. A 5476-bp retrotransposon, named OSCOPIA2 in the GIRI database, was identified in rice that has ∼70% identity with FRetro64 but the 162–163-bp LTRs share no similarity with LTRs of FRetro64 or the other four retroelements (ZM64, Sor64, Sugar64 and Swit64). None of these five potential autonomous elements share similarity with the SMARTs from their respective genomes.

In order to determine the evolutionary relationship between FRetro64 and other reported LTR retrotransposons, a phylogenetic tree was build based on conserved RT domains of the retroelements (Figure S3). FRetro64, ZM64, Sor64, Sugar64 and OSCOPIA2 were grouped into same clade indicating that these five retrotransposons were likely derived from an ancestral element.

## Discussion

### A novel retrotransposon conserved across the grass family

We report a new LTR retrotransposon (FRetro129) that is only 292 bp in length and is the smallest LTR retrotransposon reported thus far. FRetro129 does not encode any protein which indicates that the element is a nonautonomous LTR retrotransposon. In plant genomes, two nonautonomous LTR retrotransposons, LARD and TRIM, have been reported. LARD elements are large, more than 8 Kb, and are located in heterochromatin regions or chromosome arms [14], [16]; whereas, TRIM elements are smaller and distributed primarily in genic regions [12], [15]. Similar to TRIMs, we found that FRetro129 and its homologs were frequently inserted in or near genic regions. Thus, the FRetro129 (SMART) family may be classified as another group of nonautonomous LTR retrotransposon because of the following observations: First, FRetro129 is smaller than TRIMs whose sizes range from 500–900 bp and have longer TDRs, 100–350 bp. Second, TRIMs are widely distributed in both dicotyledonous and monocotyledonous species and even in the ferns; whereas, FRetro129 is restricted to the grasses. Third, TRIMs are more evolutionarily conserved than FRetro129. For example, TDRs of TRIM elements in rice and Arabidopsis show 80–90% sequence similarity [12]. Although, FRetro129 elements can have more than 80% sequence identity with copies from other grass genomes, FRetro129 elements from the same genome can be quite divergent. Some FRetro129 elements in *O. brachyantha* share less 60% sequence identity. Fourth, TRIM homologous fragments have been found in the mitochondrial genome [12]. We conducted BLASTN searches against chloroplast and mitochondrial genomes of plants including rice, wild rice, maize, sorghum, wheat, barley, *Brachypodium* and Arabidopsis and found no FRetro129 elements.

### The origin and amplification of SMARTs

So far, very little is known about the origin of retrotransposons. Phylogenetic analyses based on conserved sequences of various retrotransposons indicated that LTR retrotransposons likely originated from the fusion of a DNA transposon and a non-LTR retrotransposon and may have been present in early eukaryotes [1], [43], [44]. In plants, some LTR retrotransposons have been found in both dicotyledonous and monocotyledonous species and even in the ferns [12], [15], [45]. Thus, these LTR retrotransposons must have existed before the divergence of dicotyledonous and monocotyledonous plants (about 200 MYA). SMARTs are present not only in *O. brachyantha* and other rice species but also in many genomes of the grass family. This indicates that SMARTs were present before the radiation of the grass, ∼50–80 MYA [33], [34].

The phylogenetic tree of FRetro129 and its homologs showed intermingling of SMART elements from different genomes suggesting multiple ancient lineages of SMARTs in the grass family. Nearly all the insertion sites of SMARTs from rice, sorghum and maize that were grouped into a same subfamilies were in introns or single-copy regions. This may result in a decelerated mutation rate relative to other elements resulting in the intermingling of elements from diverged genomes. It should be noted that two complete elements from *O. sativa* showed more than 95% sequence identity with the elements from *O. brachyantha* which last shared an ancestor with *O. sativa* about 7–9 MYA. Given that retrotransposons are thought to evolve more rapidly than genes [36], we cannot rule out the possibility of horizontal transfer of the small retrotransposons within the grass family. Horizontal transfer has been reported for both Mutator-like elements (MULEs) and LTR retrotransposons within the rice genus and other genera in the grass family [46], [47]. In this study, no FRetro129 homologs were found in genomes outside the grass species. It is possible, however, that ancient homologs of FRetro129 were either lost or are highly diverged in these genomes.

We estimated the insertion times of SMARTs and found very recent insertions (0 MYA) in rice and other genomes, such as sorghum, maize and switchgrass. By comparing two subspecies of rice, *Indica* and *Japonica*, we found at least six insertions that occurred after the divergence of the two subspecies, 0.2–0.4 MYA [36], [37]. All these results suggest that the SMARTs were recently mobilized and may yet be active in some genomes of the grass family. Transposons activity is suppressed by the genome defense mechanisms including DNA methylation and siRNA silencing [28], [29], [41]. We identified more than 400 distinct sRNAs from rice that matched SMARTs. To determine if SMARTs were methylated, we searched a DNA methylation database for rice [48] with 33 complete small retroelements (total size of 8502 bp) and found that the small retroelements had 90 exact matches whereas the 6 randomly selected genes (total size of 9402 bp) had only 14 hits (data not shown). However, 14 complete SMART elements had no methylated DNA matches. Furthermore, we also searched undermethylated DNA sequences from sorgum [49] and identified 15 putatively unmethylated SMART sequences (Table S3). These results suggest that some SMARTs are methylated or silenced by sRNAs but that some SMARTs escape suppression and may be active.

FRetro129 does not have a coding region, thus its movement must catalyzed by a retrotransposase encoded by another autonomous transposon(s) in the genome. Nonautonomous transposons share sequence similarity with their autonomous partners in some regions such as in LTRs or TIRs [16], [50]. FRetro64 is a putative autonomous retrotransposon for FRetro129 based on the following observations: 1) FRetro64 shares 96% sequence identity with the internal region of FRetro129; 2) LTRs of both FRetro64 and FRetro129 contain an 11-bp conserved motif; 3) FRetro64 is the only element with detectable sequence similarity to FRetro129 in the *O. brachyantha* genome. Surprisingly, no sequences show significant similarity with homologs of FRetro129 in rice, sorghum and maize. The LTRs of putative autonomous retrotransposons in rice, sorghum and maize identified by FRetro64 also display no similarity with the LTRs of FRetro64. These results indicate a complex interaction between SMARTs and their autonomous elements. It was reported that autonomous and nonautonomous elements may show no or low sequence similarity. For example, 4 distinct SINEs lineages were found in the mouse genome, however, none shares sequence similarity with the LINEs that are thought to catalyze retrotransposition [51]. Thus, we cannot rule out the possibility that the autonomous element may show no detectable sequence similarity with FRetro129 or is no longer present in the genome. Further work is needed to clearly determine the retrotransposase that catalyzes the movement of FRetro129.

LTRs are important components of retrotransposons because they possess regulatory signals for transcription that include promoter sites (unique 3′ RNA, U3), polyadenylation sequences (repeated RNA, R) and transcript terminator signals (unique 5′ RNA, U5) [1]. The LTRs of FRetro129 elements contain TATAAAA, a typical TATA promoter box motif. However, no polyadenylation or termination sequences were identified in the LTR regions (Figure S4). It is not clear if the FRetro129 elements use the promoters in their LTR regions and co-opt termination signals from downstream sequences in order to amplify, or if the elements rely on the replication of host genes as many SMART elements were found in introns or UTRs.

### The evolutionary impact of SMARTs

We found that the small retrotransposons are often located within or near genes and are most frequently found in introns and UTR regions. Previous studies have showed that miniature inverted-repeat transposable elements (MITEs) are frequently associated with genes and are often found in introns [52]–[55]. Thus, the insertion patterns of the SMARTs are similar to that of MITEs, despite the fact that they are members of a different transposon class and move via distinct transposition mechanisms. Since introns are removed by RNA splicing machinery before translation [56], [57], intronic insertions may not affect the gene structure. In fact, comparative analyses between the genes with insertions and orthologous and/or paralogous genes indicated that most insertions do not change the gene structure.

However, we did find 11 genes in which SMARTs may affect splicing as the genes with insertions show different structures from either the orthologous or paralogous genes. Therefore, SMARTs do at some frequency affect gene structure and may play a role in evolution of genomic diversity and novelty. We cannot, however, rule out the possibility that other factors may also result in altered gene structures.

One role of sRNAs in eukaryotes is to suppress transposons by epigenetic mechanisms [58] as evidenced by increased transposition when DNA methylation is impaired or when the biogenesis of small interfering RNAs (siRNAs) is altered [29], [41], [59]. In this study, we identified more than 400 sRNAs that perfectly match SMARTs, some of which appear to target the expressed genes (Figure 8). Gene regulation mediated by siRNAs targeting intronic transposons has been reported in both plant and human [60], [61]. Thus, sRNAs may be involved not only in silencing of SMARTs but also in gene regulation in or near genes where SMARTs reside. qRT-PCR results indicated that an intergenic insertion of a SMART could increase the expression level of flanking genes five to thirteen fold. Because the insertion was located about 1 kb region from both flanking genes, it is possible the element may be inserted into a regulatory region and is acting as a promoter or enhancing region. However, it is not clear how up regulation of these genes was achieved and whether sRNAs are involved in the regulation.

Formation of solo-LTRs is thought to be an important way to reduce the genome size though the mechanism is poorly understood. It has been reported that LTR sizes of the retrotransposons may affect the likelihood of recombination between LTRs. Thus, retrotransposon families with longer LTRs show higher ratios of solo-LTR to complete elements than those with shorter LTRs [25]. Another outstanding question is how many nucleotides are required for the formation of a solo-LTR via illegitimate recombination? In bacteria, at least 20 bp are required and 50 to 100 bp in yeast [62], [63], but nothing is known in plants. We identified solo LTRs of SMARTs in many of the species, including rice, maize and sorghum. The ratios of complete element to solo-LTR ranged from 50∶1 in sorghum to 3.4∶1 in *O. brachyantha*. Our results indicate that retrotransposons with small LTRs can generate solo-LTRs and that the genomic environment may affect the formation of solo-LTR. Since the LTRs are 85 bp, that sequences as short as 85 bp are enough for homologous recombination.

Although the formation of solo-LTR is an efficient way to reduce the genome sizes, retrotransposons may not benefit from this activity as there is no demonstrated way to amplify solo LTRs. Thus, the fate for solo-LTRs is that they either accumulate mutations and became genomic fossils or, in some cases, are recruited as gene components. SMARTs are small and frequently located within intronic regions, but these elements can be amplified and maintained in the genomes over long evolutionary timeframes. We hypothesize that it may represent another strategy for plant genomes and LTR retrotransposons to co-exist and co-evolve.

### Practical utilization of SMARTs

Transposons have been widely used as insertional mutagens in plant functional genomics. For example, numerous mutants in maize have been generated using the Mutator and Ac/Ds transposons tagging systems [64], [65]. In rice, an active LTR retrotransposon, *Tos*17, has been used to create ∼50,000 *Tos*17-insertion lines [66], [67]. The identification of SMARTs and their recent insertions into some grass genomes may provide a tool for gene tagging in the grass species. Our results from rice, sorghum and maize indicated that SMARTs preferentially insert into genic regions, especially introns. Some SMART insertions were also in UTRs or exons. Moreover, we found 116 ESTs or cDNAs from 16 grass genomes that contain SMARTs (Table S4). This suggests that SMARTs are expressed and that SMARTs may be a potential mutagen for functional genomics in plants, particularly grass species.

## Materials and Methods

### Materials

Seeds of a total of 22 genotypes from different organisms, including rice (*O. sativa*), wild rice species, maize, barley, sorghum and other genomes, were provided by different laboratories or were collected by our laboratory (Table S5). All seeds were planted and grown in the greenhouse at Purdue University.

### Genome sequences

The draft genome sequence of *O. brachyantha* was downloaded from the website at ftp://Oryza_FF:ydq2eysc15x@ftp.genomics.org.cn. The genome sequences of Nipponbare and 93-11 were obtained from the International Rice Genome Sequencing Project (IRGSP) website (http://rgp.dna.affrc.go.jp/E/IRGSP/index.html) and the BGI website (http://rice.genomics.org.cn/rice/link/download.jsp), respectively. Other genome sequences, including maize, sorghum, *Brachypodium*, Arabidopsis, papaya, soybean, wine grape and poplar, were downloaded from the PlantGDB website (http://www.plantgdb.org/prj/GenomeBrowser).

### Sequence characterization of SMARTs

In order to identify SMARTs in the genome sequence of *O. brachyantha*, the LTR-Finder program [32] was used with default parameters except that we set a 50 bp of minimum LTR length and 100 bp of minimum distance between LTRs. The output “LTR retrotransposons” were then manually inspected to rule out the incorrectly predicted sequences and to determine the exact boundaries of retroelements.

To detect homologous elements of FRetro129 in related genomes, the 27 complete members of FRetro129 family were used to screen the whole genome sequences from Nipponbare, 93-11, maize, sorghum, *Brachypodium*, Arabidopsis, papaya, soybean, grape and poplar with the RepeatMasker program (http://www.repeatmasker.org) using default parameters with the “nolow” option. We also set a cutoff score greater than 250 and hit sequence length longer than 50 bp. Additionally, the TE library of FRetro129 was utilized to search against BAC end sequences (BES) database of 11 wild rice species including *O. glaberrima*, *O. nivara*, *O. rufipogon*, *O. punctata*, *O. minuta*, *O. officinalis*, *O. alta*, *O. australiensis*, *O. granulate*, *O. ridleyi* and *O. coarctata* (http://www.omap.org) using RepeatMasker with same settings as above. Furthermore, the FRetro129 elements were used individually as query to conduct BLASTN searches against database in GenBank including nonredundant (nr), reference mRNA sequences (refseq_rna), expressed sequence tags (ESTs), genomic survey sequences (gss), high-throughput genomic sequences (htgs) and whole-genome shotgun reads (wgs). The significant hits (E value<10^−5^) were careful inspected to examine the boundaries of each element and target site duplications (TSD). In this study, the homologous elements are the elements that share similar structures with the FRetro129 element and can be recognize by the FRetro129 sequences using BLASTN and RepeatMasker programs. The full-length or complete elements are sequences that have two relatively intact LTRs flanked by TSDs. solo-LTRs indicate elements that contain an intact LTR sequences flanked by TSDs.

5′ and 3′ TDR sequences of the small retransposons were aligned and used to estimate insertion time of complete retrotransposons. The insertion times (T) were calculated using the formula: T = K/2r where K is average number of substitutions per aligned site and r means an average substitution rate which is 1.3×10^8^ substitutions per synonymous site per year as suggested by Ma and Bennetzen [36].

### Disruption of gene structures by insertion of SMARTs

SMARTs in Nipponbare and the 1.5 kb flanking sequences for each side were used to search against the rice genome annotation project website (http://rice.plantbiology.msu.edu) to find gene structures. To predict the sequences in sorghum and maize, 20 kb of flanking sequence (10 kb on each side of the transposon) were analyzed by the FGENESH (http://linux1.softberry.com) and the GeneMark.hmm (http://opal.biology.gatech.edu/GeneMark). Additionally, all flanking sequences and the transposons also were used as queries for BLASTN and BLASTX searches against cDNAs and proteins in GenBank.

In order to analyze the effect of SMART insertion on gene structures, we used the reference gene sequences to search against the nonredundant (nr) and reference mRNA sequences database in GenBank. In addition, we set multiple criteria for comparisons. 1) All predicted exon-intron structures of the genes must be supported by cDNA sequences that species that covers the entire coding regions; 2) Although full gene structures were analyzed, we focused primarily on the two exons adjacent to the SMART; and 3) The orthologous and paralogous genes should not have SMART insertions. The analysis was very conservative: if the two adjacent exons shared the same splicing sites as the orthologous or paralogous sequences, this was considered to have no effect on gene structures, even other parts of the genes had differences.

### Phylogenetic analysis

In order to determine the evolutionary relationship between FRetro129 and the homologous elements, 200 complete elements were used to build a phylogenetic tree, which includes 27 elements from *O. brachyantha*, 23 elements from Nipponbare, 13 elements from 93-11, 14 elements from the wild rice species (1 in each frp, *O. barthii*, *O. glaberrima*, *O. punctata*, *O. ridleyi*, *O. australiensis* and *O. coarctata*, 2 from *O. alta* and *O. granulate*, 4 from *O. minuta*), 39 maize elements, 41 sorghum elements, 30 elements from *Brachypodium*, and 6 and 7 elements from sugarcane and switch grass, respectively. All these sequences were aligned using the CLUSTAL W program [68] with default options. The phylogenetic tree was generated using neighbor-joining method in the MEGA 4 program [69]. The analysis was based on 1000 bootstrap replicates, using the nucleotide: maximum composite likelihood model. We also constructed another phylogenetic tree based on conserved RT domains of FRetro64 and other retrotransposons using same method as above and our previous report [23]. The sequences used to build the phylogenetic trees are listed in Table S6 and the sequences of FRetro129 and FRetro64 have been deposited in GenBank under the accession numbers JN806223 and JN806224.

### Quantitative RT-PCR (qRT- PCR)

Total RNA was extracted from sheaths and leaves of 4-week old plants of Nipponbare (*Japonica*) and 93-11 (*Indica*) using the TRIZOL Reagent (Invitrogen, Carlsbad, CA). 5 µg total RNA from each sample was treated with the RQ1 RNase-free DNase (Promega, Madison, WI) and converted into single strand cDNA with reverse transcriptase (Invitrogen, Carlsbad, CA).

qRT-PCR assays were performed in triplicate and repeated on three independent biological samples with 2× SYBR® Green PCR Master Mix buffer (Applied Biosystems, Foster City, CA) in a 20- µL volume containing 1 µL cDNA and 0.5 µM of each forward and reverse primers (Table S7). The reactions were run on an Applied Biosystems 7500 Fast Real-Time PCR system (Applied Biosystems, Foster City, CA). The data were analyzed according to the previous articles [70], [71]. Briefly, the qRT-PCR data were used to calculate the average cycle threshold (Ct) values and the standard deviations for each gene/tissue combinations. The ΔCt values for each of target rice genes were calculated by the formula: Ct value of target gene - Ct value of actin gene. In order to estimate the effect of the TE insertions on gene expression, the relative expression level of each of TE related genes was described as the percentage of the orthologous gene which no TE inserted in or near.

### Southern blot analysis

6 µg plant genomic DNAs were digested by *Eco*R I (New England, Ipswich, MA) at 37°C for overnight. The digested DNAs were separated by electrophoresis on a 0.8% (w/v) agarose gel at 45 v for 12 h and transferred onto a Hybond N^+^ membrane (Amersham Biosciences, Piscataway, NJ). The genomic DNA of *O. brachyantha* was used to amplify PCR products for southern blot with the following primers: FRetro129 (Forward: 5′-GGAGTGTATAAAGTGAATTGCC-3′ and Reverse: 5′- CATGCACCAGCCAGTTGCACC -3′); FRetro129-19 (Forward: 5′- CACAGAGTGAATTACCTGTTTTTCC -3′ and Reverse: 5′- CACCAGCAAGTTGCACCTAA -3′); FRetro129-108 (Forward: 5′- ACGTGAATTGACCGCCTTTA -3′ and Reverse: 5′- GCTTAAGCTGGTGAGCAAAG -3′); FRetro129-116 (Forward: 5′- TGAATTACCTGCTTTTTCCTATCA-3′ and Reverse: 5′- ACCAGCCAGTTGCACCTAAA -3′). A mixture of the above 4 PCR products was used as a probe to detect the presence of FRetro129 in different plant genomes. The PCR fragment was labeled with ^32^P-dCTP using the rediprime II random prime labeling system (Amersham Biosciences, now part of GE Healthcare, Little Chalfont, England) according to the manufacturer's instructions. Hybridized were performed at 55°C for overnight and washed in 1.5× SSC solution for 30 min and in 1× SSC for 20 min. The membrane was exposed on a Fuji-image plate and the hybridization signals were captured using a Fujifilm FLA-5100 multifunctional scanner.

## Supporting Information

Figure S1 **Sequence alignment of FRetro129 and the elements from other genomes.** The LTRs and internal regions of 18 SMATs were marked by arrows and vertical lines, respectively.
(TIF)

Figure S2 **Alignment of a rice small RNA, osa-smRNA15336, and the SMARTs from different genomes.**
(TIF)

Figure S3 **A phylogenetic tree of different Ty1-copia LTR retrotransposons.** The phylogenetic tree was generated based on the conserved RT domains of 42 Ty1-copia like retrotransposons from *O. brachyantha* and other organisms.
(TIF)

Figure S4 **LTR sequences of FRetro129 elements.** The TATA box is marked by the rectangle and arrows indicate the 4-bp inverted repeats (TGTT…AACA) of the LTRs.
(TIF)

Table S1 Gene structure comparisons between reference genes in sorghum and maize and their orthologous genes. Sorghum genes named with Sb, maize genes with LOC and rice genes with Os, Os02g19150 gene model is from the MSU rice genome annotation project, others are from GenBank.
(DOCX)

Table S2 A list of PAREs related to SMARTs.
(DOCX)

Table S3 A list of methylation filtered (undermethylated) sequences containing SMARTs in Sorghum.
(DOCX)

Table S4 Identification of transcription sequences in grass.
(DOCX)

Table S5 A list of plants used in this study.
(DOCX)

Table S6 GenBank accession numbers of annotated transposons used in this study.
(DOCX)

Table S7 List of primers for qRT-PCR.
(DOCX)
