# Supplementary figures and images for: A Highly Conserved, Small LTR Retrotransposon that Preferentially Targets Genes in Grass Genomes
Source: PLoS One. 2012 Feb 16;7(2):e32010. doi: 10.1371/journal.pone.0032010 (PMC3281118; doi:10.1371/journal.pone.0032010)

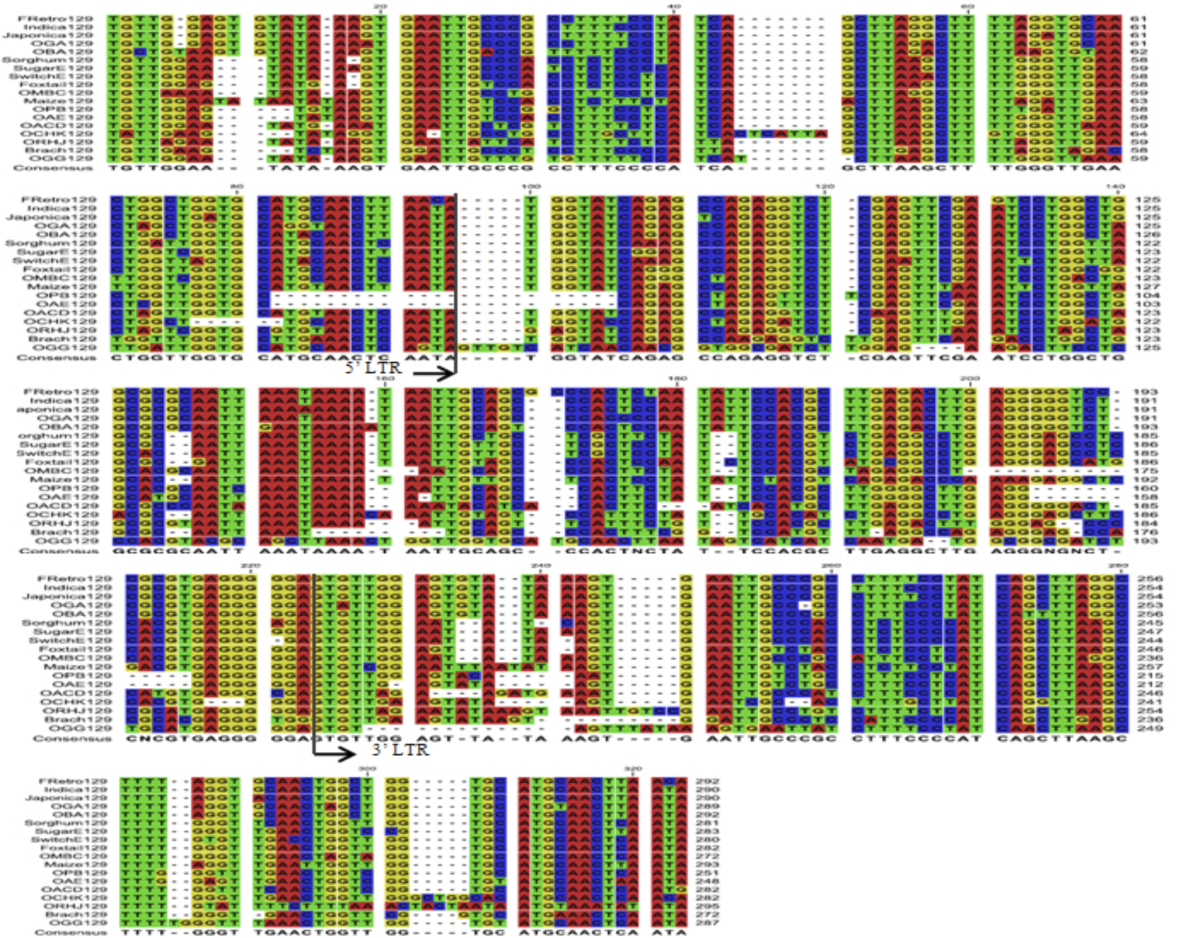

Supplement: Figure S1 — Sequence alignment of FRetro129 and the elements from other genomes. The LTRs and internal regions of 18 SMATs were marked by arrows and vertical lines, respectively. (TIF) [file pone.0032010.s001.tif]

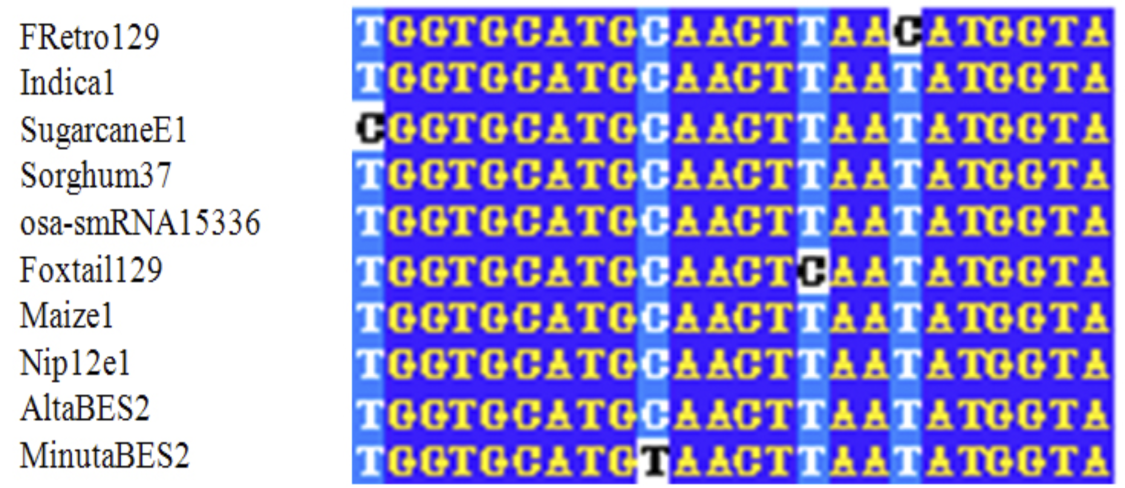

Supplement: Figure S2 — Alignment of a rice small RNA, osa-smRNA15336, and the SMARTs from different genomes. (TIF) [file pone.0032010.s002.tif]

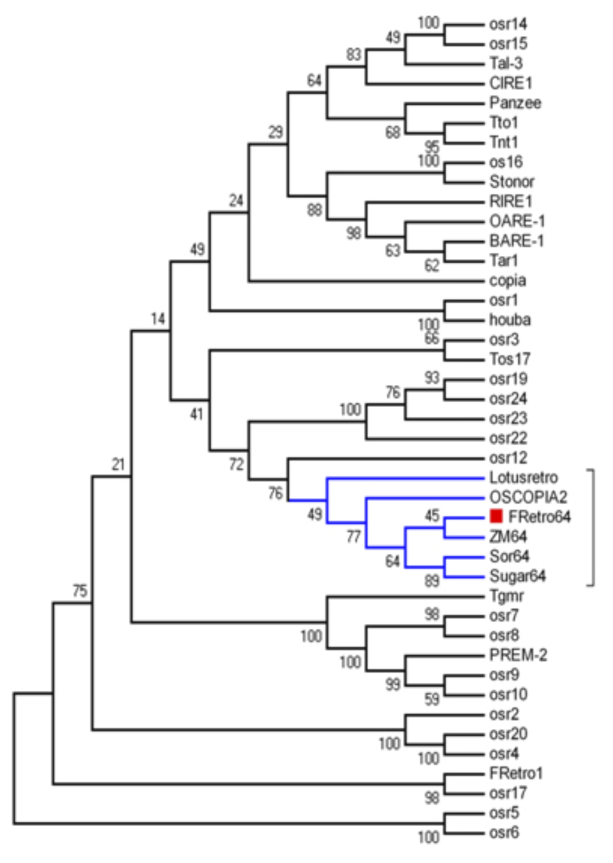

Supplement: Figure S3 — A phylogenetic tree of different Ty1-copia LTR retrotransposons. The phylogenetic tree was generated based on the conserved RT domains of 42 Ty1-copia like retrotransposons from O. brachyantha and other organisms. (TIF) [file pone.0032010.s003.tif]

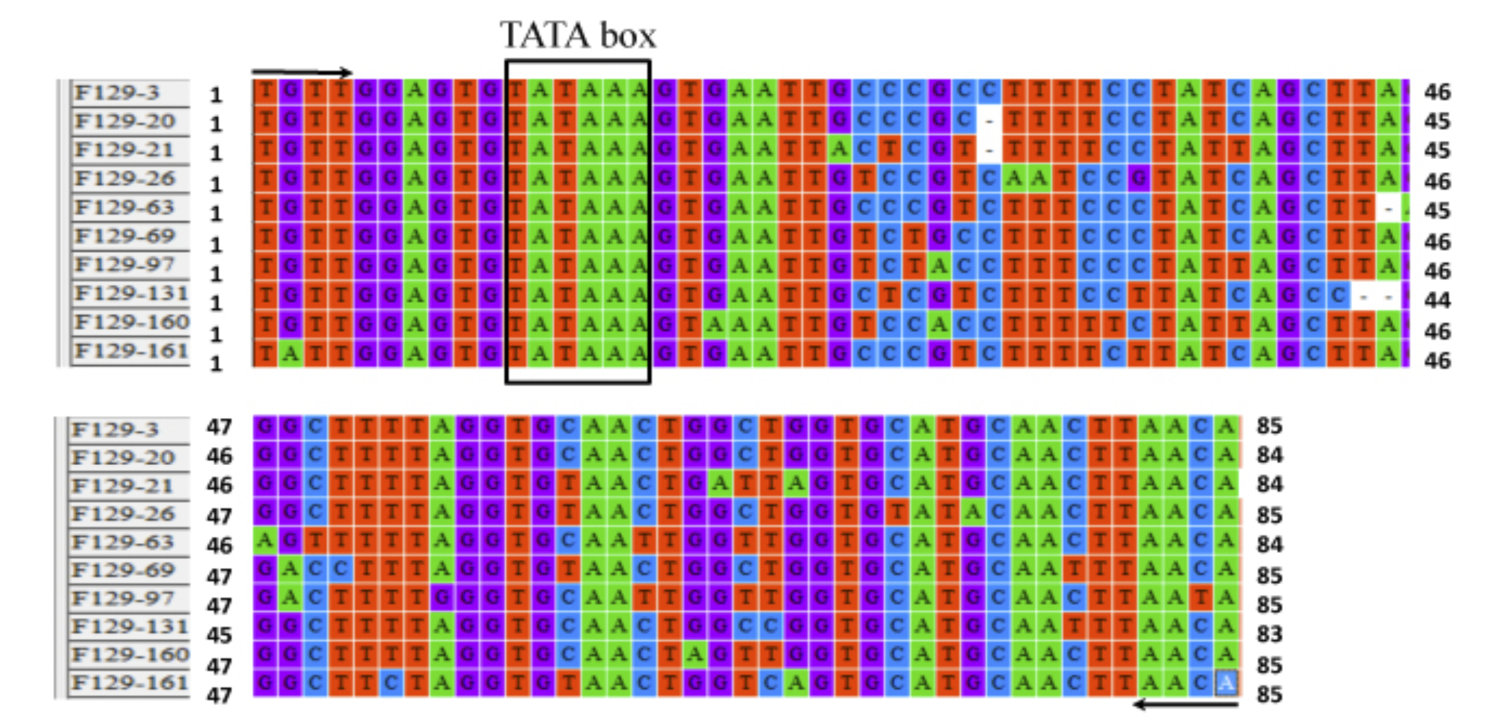

Supplement: Figure S4 — LTR sequences of FRetro129 elements. The TATA box is marked by the rectangle and arrows indicate the 4-bp inverted repeats (TGTT…AACA) of the LTRs. (TIF) [file pone.0032010.s004.tif]
