# Supplementary material for: A Highly Conserved, Small LTR Retrotransposon that Preferentially Targets Genes in Grass Genomes
Source: PLoS One. 2012 Feb 16;7(2):e32010. doi: 10.1371/journal.pone.0032010 (PMC3281118; doi:10.1371/journal.pone.0032010)
Supplement: Table S1 — Gene structure comparisons between reference genes in sorghum and maize and their orthologous genes. Sorghum genes named with Sb, maize genes with LOC and rice genes with Os, Os02g19150 gene model is from the MSU rice genome annotation project, others are from GenBank. (DOCX) [file pone.0032010.s005.docx]

| Genes | mRNAs | Gene sizes (bp) | Locations of elements | Orthologous gene 1 | | Orthologous gene 2 | |
| --- | --- | --- | --- | --- | --- | --- | --- |
|  |  |  |  | Genes | mRNAs | Genes | mRNAs |
| Sb04g011760 | XM_002453688 | 11,192 | 5326-6049 (9^th^ intron) | LOC100281116 | NM_001154035 | Os02g19150 | NM_001053135 |
| Sb06g018698 | XM_002446554 | 3,797 | 1288-1564 (2^ed^ intron) |  |  | Os01g0867100 | NM_001185738 |
| Sb06g024520 | XM_002446852 | 14,544 | 8060-8314 (1^st^ intron)  10548-10825 (4^th^ intron) | LOC100216704 | NM_001143109 | Os04g0549600 | NM_001060020 |
| Sb06g024850 | XM_002448253 | 9,755 | 569-768 (1^st^ intron) |  |  | Os02g0663300 | NM_001054191 |
| Sb06g025390 | XM_002448292 | 19,391 | 7564-7844 (17th intron) |  |  | Os04g0563801 | NM_001187114 |
| Sb08g001630 | XM_002441700 | 5,816 | 4257-4538 (6th intron) | LOC100278822 | NM_001151972 | Os12g0126800 | NM_001072583 |
| Sb09g023240 | XM_002439904 | 8,067 |  | [LOC100191628](http://www.ncbi.nlm.nih.gov/sites/entrez?db=gene&cmd=search&term=100191628&RID=SB5E3R9D013&log$=geneexplicitnucl&blast_rank=4) | NM_001137057 | Os05g0474600 | NM_001062361 |
| LOC100281744 | NM_001154664 | 5,425 | 3960-4238 (5’UTR) | Sb01g038740 | XM_002468007 | Os03g0286200 | NM_001056307 |
| LOC100274016 | NM_001148401 | 7,648 | 5322-5616 (9^th^ intron) | Sb02g030440 | XM_002462654 | Os09g0525700 | NM_001070257 |
| LOC100192992 | NM_001138163 | 13,036 | 7556-7835 (6^th^ intron) |  |  | Os06g0221100 | NM_001063710 |
