# Supplementary material for: A Highly Conserved, Small LTR Retrotransposon that Preferentially Targets Genes in Grass Genomes
Source: PLoS One. 2012 Feb 16;7(2):e32010. doi: 10.1371/journal.pone.0032010 (PMC3281118; doi:10.1371/journal.pone.0032010)
Supplement: Table S2 — A list of PAREs related to SMARTs. (DOCX) [file pone.0032010.s006.docx]

| Number | PARE | Sequence | Size (nt) | Hits in Nipponbere |
| --- | --- | --- | --- | --- |
| 1 | Os01g53840-247 | cagttgcacctaaaagccta | 20 | 25 |
| 2 | Os01g53840-248 | ttgcacctaaaagcctaagc | 20 | 25 |
| 3 | Os01g53840-249 | tgcacctaaaagcctaagct | 20 | 25 |
| 4 | Os01g53840-250 | aactggctggtgcatgcaac | 20 | 25 |
| 5 | Os01g53840-251 | gcacctaaaagcctaagctg | 20 | 25 |
| 6 | Os01g53840-252 | taaaagcctaagctgatagg | 20 | 30 |
| 7 | Os01g53840-253 | aggtgcaactggctggtgca | 20 | 24 |
| 8 | Os01g53840-254 | tttaggtgcaactggctggt | 20 | 25 |
| 9 | Os01g53840-255 | ttttaggtgcaactggctgg | 20 | 25 |
| 10 | Os01g53840-256 | gcttttaggtgcaactggct | 20 | 24 |
| 11 | Os01g53840-257 | ctaagctgatagggaaaggc | 20 | 16 |
| 12 | Os01g53840-258 | agctgatagggaaaggcggg | 20 | 14 |
| 13 | Os01g53840-259 | gctgatagggaaaggcgggc | 20 | 14 |
| 14 | Os01g53840-261 | gatagggaaaggcgggcaat | 20 | 21 |
| 15 | Os01g53840-262 | ctttccctatcagcttaggc | 20 | 31 |
| 16 | Os01g53840-263 | gcgggcaattcactttatac | 20 | 27 |
| 17 | Os01g53840-264 | gggcaattcactttatacac | 20 | 42 |
| 18 | Os01g53840-265 | cactttatacactccaacac | 20 | 27 |
| 19 | Os02g50340-332 | ttttagatgtaactggctgg | 20 | 1 |
| 20 | Os02g50340-333 | agaggtcttgagttcgaatc | 20 | 2 |
| 21 | Os02g50340-334 | ttattttatttaattgcgcg | 20 | 25 |
| 22 | Os02g50340-335 | ccactccaatattccacgct | 20 | 24 |
| 23 | Os02g50340-336 | tattccacgcttgagacttg | 20 | 31 |
| 24 | Os02g50340-337 | gcttgagacttgagggggag | 20 | 2 |
| 25 | Os02g50340-338 | gcgtggaatattggagtggg | 20 | 21 |
| 26 | Os02g50340-339 | ttgagggggagtgttggagt | 20 | 3 |
| 27 | Os02g50340-340 | ccccctcaagtctcaagcgt | 20 | 25 |
| 28 | Os02g56970-2 | attcttatagcttaagttct | 20 | 1 |
| 29 | Os02g56970-3 | ttggaatataaagtgaattg | 20 | 28 |
| 30 | Os02g56970-4 | acacaattcctgaaaattgc | 20 | 1 |
| 31 | Os03g24610-27 | cctaagctgatagggaaaga | 20 | 16 |
| 32 | Os04g03796-447 | gcaccgaccatttcaaccca | 20 | 2 |
| 33 | Os04g03796-448 | tgggcaattcactttatatt | 20 | 8 |
| 34 | Os04g03796-449 | gcaattcactttatattcta | 20 | 3 |
| 35 | Os04g03796-450 | ttcactttatattctaacac | 20 | 2 |
| 36 | Os04g03796-451 | tttatattctaacactcccc | 20 | 1 |
| 37 | Os04g03796-452 | atattctaacactcccccta | 20 | 1 |
| 38 | Os04g03796-453 | attctaacactccccctaac | 20 | 1 |
| 39 | Os04g03796-454 | tctaacactccccctaacgc | 20 | 1 |
| 40 | Os04g03796-455 | cctaacgcgaggccccctca | 20 | 1 |
| 41 | Os04g03796-456 | aacgcgaggccccctcaagc | 20 | 1 |
| 42 | Os04g03796-457 | ccccctcaagcctctaacgt | 20 | 1 |
| 43 | Os04g03796-458 | ctctaacgtggaataagaag | 20 | 1 |
| 44 | Os04g03796-459 | cgtggaataagaagtgggca | 20 | 1 |
| 45 | Os04g03796-460 | gtgggcagcaatttttattt | 20 | 1 |
| 46 | Os04g03796-461 | ctagccaagattcgaactcg | 20 | 1 |
| 47 | Os04g03796-462 | agccaagattcgaactcgag | 20 | 1 |
| 48 | Os04g03796-464 | gatgaggaaaagtgagcaat | 20 | 1 |
| 49 | Os04g03796-465 | ttccaacacatacatgcctt | 20 | 1 |
| 50 | Os06g51420-24 | taagcttttgagttgaactg | 20 | 3 |
| 51 | Os06g51420-25 | agcttttgagttgaactggt | 20 | 3 |
| 52 | Os06g51420-26 | acctctggctctgataccat | 20 | 20 |
| 53 | Os07g09520-17 | tttggctctgatactactat | 20 | 1 |
| 54 | Os07g09520-18 | tttgagttcgaatcctgact | 20 | 1 |
| 55 | Os07g09520-19 | ttgagttcgaatcctgactt | 20 | 1 |
| 56 | Os07g09520-20 | tcaaaacttttggctctgat | 20 | 1 |
| 57 | Os07g09520-21 | tttctcatcagcttaggtat | 20 | 1 |
| 58 | Os07g09520-22 | taagctgatgagaaaagacg | 20 | 1 |
| 59 | Os08g15060-184 | ttatattctaacaggattgt | 20 | 1 |
| 60 | Os09g25945-57 | taactggttggtgcatgcaa | 20 | 3 |
| 61 | Os09g25945-58 | aactggttggtgcatgcaac | 20 | 6 |
| 62 | Os09g25945-59 | actggttggtgcatgcaact | 20 | 5 |
| 63 | Os09g25945-60 | ttggtgcatgcaacttaata | 20 | 4 |
| 64 | Os09g25945-61 | gtgcatgcaacttaatatgg | 20 | 21 |
| 65 | Os09g25945-62 | acttaatatggtattagagc | 20 | 2 |
| 66 | Os09g25945-63 | ataccatattaagttgcatg | 20 | 31 |
| 67 | Os09g25945-64 | aataattgcagcccactcca | 20 | 17 |
| 68 | Os09g25945-65 | ttattttatttaattgcgcg | 20 | 25 |
| 69 | Os09g25945-66 | ttgcagcccactccaatatt | 20 | 17 |
| 70 | Os09g25945-67 | caattattttatttaattgc | 20 | 29 |
| 71 | Os09g25945-72 | aagctgatagggaaagacgg | 20 | 8 |
| 72 | Os09g25945-73 | ctaagctgatagggaaagac | 20 | 10 |
| 73 | Os09g25945-84 | cagttgcacctaaaagccta | 20 | 25 |
| 74 | Os11g02720-56 | ccccctcacgcgagaccccc | 20 | 15 |
| 75 | Os11g02720-57 | cctcacgcgagaccccctca | 20 | 16 |
| 76 | Os11g02720-66 | gagacctctggctttgatac | 20 | 2 |
| 77 | Os11g02720-67 | tcgagttcgaatcctggctg | 20 | 21 |
| 78 | Os11g02720-68 | acctctggctttgataccat | 20 | 2 |
| 79 | Os11g02720-69 | aggtctcgagttcgaatcct | 20 | 27 |
| 80 | Os11g02720-70 | gctttgataccatattaagt | 20 | 2 |
