# Supplementary material for: A Highly Conserved, Small LTR Retrotransposon that Preferentially Targets Genes in Grass Genomes
Source: PLoS One. 2012 Feb 16;7(2):e32010. doi: 10.1371/journal.pone.0032010 (PMC3281118; doi:10.1371/journal.pone.0032010)
Supplement: Table S3 — A list of methylation filtered (undermethylated) sequences containing SMARTs in Sorghum. (DOCX) [file pone.0032010.s007.docx]

| Accession number | Size  (bp) | Matched sequence region | Matched  SMARTs regions | Note |
| --- | --- | --- | --- | --- |
| CW238142 | 735 | 251-518 | 15-290 |  |
| CW350020 | 748 | 424-144 | 1-292 | Element (TSD:atctg) |
| CW044126 | 610 | 246-527 | 1-292 | Element (TSD:gtttt) |
| CW262000 | 613 | 241-514 | 1-292 | Element (TSD:atgag) |
| CW123601 | 578 | 233-513 | 1-292 | Element (TSD:taaat) |
| BZ344845 | 543 | 433-158 | 1-292 | Element (TSD:ctcat) |
| CW455628 | 485 | 391-112 | 1-292 | Element (TSD:ctagt) |
| CW439585 | 716 | 524-244 | 1-292 | Element (TSD:atagt) |
| CW326355 | 683 | 464-196 | 15-290 |  |
| CW501829 | 668 | 667-436 | 35-290 |  |
| CW486171 | 733 | 545-723 | 1-188 |  |
| CW401124 | 560 | 191-358 | 1-173 |  |
| CW496616 | 685 | 117-3 | 15-129 |  |
| CW399802 | 653 | 642-490 | 128-290 |  |
| CW046321 | 604 | 529-256 | 1-292 | Element |
