# Supplementary material for: A Highly Conserved, Small LTR Retrotransposon that Preferentially Targets Genes in Grass Genomes
Source: PLoS One. 2012 Feb 16;7(2):e32010. doi: 10.1371/journal.pone.0032010 (PMC3281118; doi:10.1371/journal.pone.0032010)
Supplement: Table S4 — Identification of transcription sequences in grass. (DOCX) [file pone.0032010.s008.docx]

| Genome | Number of transcription sequences (EST or cDNA) |
| --- | --- |
| Rice (*O. sativa*) | 16 |
| Maize (*Zea mays*) | 20 |
| Sugarcane (*Saccharum*) | 8 |
| Switchgrass (*Panicum virgatum*) | 50 |
| Sorghum | 3 |
| Wheat (*Triticum aestivum* ) | 1 |
| Barley (*Hordeum vulgare* ) | 1 |
| Pearl millet (*Pennisetum glaucum*) | 1 |
| Perennial Triticeae | 1 |
| [Meadow fescue (*Festuca pratensis*)](http://www.illinoiswildflowers.info/grasses/plants/mdw_fescue.htm) | 5 |
| Perennial ryegrass | 2 |
| Brachypodium distachyon | 2 |
| Tall fescue (*Festuca arundinacea*) | 3 |
| Bluebunch wheatgrass (*Pseudoroegneria spicata*) | 1 |
| Canada wild rye (*Elymus wawawaiensis*) | 1 |
| Phyllostachys edulis | 1 |
|  |  |
| Total | 116 |
