# Supplementary material for: A Highly Conserved, Small LTR Retrotransposon that Preferentially Targets Genes in Grass Genomes
Source: PLoS One. 2012 Feb 16;7(2):e32010. doi: 10.1371/journal.pone.0032010 (PMC3281118; doi:10.1371/journal.pone.0032010)
Supplement: Table S5 — A list of plants used in this study. (DOCX) [file pone.0032010.s009.docx]

| Genomes | Note | Provided by |
| --- | --- | --- |
| Nipponbare | *O. sativa. japonica* | Jackson’s lab, University of Georgia (UGA). USA |
| 93-11 | *O sativa .indica* | Jianxin Ma, Purdue University |
| *O. glaberrima* | AA genome | Jackson lab, UGA |
| *O. nivara* | AA genome | Jackson lab, UGA |
| *O. longistaminata* | AA genome | Jackson lab, UGA |
| *O. rufipogon* | AA genome | Jackson lab, UGA |
| *O. punctata* | BB genome | Jackson lab, UGA |
| *O. officinalis* | CC genome | Jackson lab, UGA |
| *O. minuta* | BBCC genome | Jackson lab, UGA |
| *O. alta* | CCDD genome | Jackson lab, UGA |
| *O. australiensis* | EE genome | Jackson lab, UGA |
| *O. brachyantha* | FF genome | Jackson lab, UGA |
| *O. granulata* | GG genome | Jackson lab, UGA |
| *O. ridleyi* | HHJJ genome | Jackson lab, UGA |
| *O. coarctata* | HHKK genome | Jackson lab, Purdue University |
| Barley-P713/CB8 |  | Patrick Hayes, Oregon State University, USA |
| Maize -B73 |  | Dan Szymanski, Purdue University |
| Wheat -Norm |  | Jinrong Xu, Purdue University |
| Sorghum-09-27 WC |  | Gebisa Ejeta, Purdue University |
| Arabidopsis |  | Dan Szymanski, Purdue University |
| Soybean-Willims 82 |  | Jackson lab, UGA |
| Tomato |  | Jackson lab, UGA |
