# Supplementary material for: A Highly Conserved, Small LTR Retrotransposon that Preferentially Targets Genes in Grass Genomes
Source: PLoS One. 2012 Feb 16;7(2):e32010. doi: 10.1371/journal.pone.0032010 (PMC3281118; doi:10.1371/journal.pone.0032010)
Supplement: Table S6 — GenBank accession numbers of annotated transposons used in this study. (DOCX) [file pone.0032010.s010.docx]

| \| Name \|  \|  \| \| --- \| --- \| --- \| | Organism | Size (bp) | TE super family | GenBank accession number | Location in the sequence |
| --- | --- | --- | --- | --- | --- | --- | --- | --- |
| Japonica129 | \| O. sativa(japonica) \| \| --- \| | 290 | unknown | AL713908 | 107838-107549 |
| Indica129 | O. sativa(indica) | 290 | unknown | AAAA02007037 | 21289-21578 |
| OGA129 | O. glaberrima | 289 | unknown | AC223443 | 53496-53784 |
| OBA129 | O. barthii | 292 | unknown | ABRL01001385 | 18835-19126 |
| OPB129 | O. punctata | 251 | unknown | CW623350 | 246-496 |
| OMBC129 | O. minuta | 271 | unknown | AC231933 | 42778-43048 |
| OACD129 | O. alta | 282 | unknown | CZ580118 | 26-307 |
| OAE129 | O. australiensis | 248 | unknown | ED890268 | 246-493 |
| OGG129 | O. granulata | 287 | unknown | DU167175 | 341-627 |
| ORHJ129 | O. ridleyi | 295 | unknown | GQ203303 | 176994-177288 |
| OCHK129 | O. coarctata | 282 | unknown | CZ856941 | 458-739 |
| Maize129 | Maize | 293 | unknown | AC210721 | 52689-52981 |
| Sorghum129 | Sorghum | 281 | unknown | NW_002994238 | 27374024-27374304 |
| Sugarcane129 | Saccharum officinarum | 283 | unknown | CA170015 | 275-557 |
| Brach129 | Brachypodium distachyon | 272 | unknown | ADDN01000039 | 145132-145403 |
| Switchg129 | Panicum virgatum | 280 | unknown | HO281922 | 97-376 |
| Foxtail129 | Setaria italica | 282 | unknown | GS106180 | 485-766 |
| Sor64 | sorghum | 5785 | Ty1-copia | AC120496 | 26285-20501 |
| Sugar64 | sugarcane | 5622 | Ty1-copia | AM403007 | 26012-31633 |
| ZM64 | maize | 4871 | Ty1-copia | AC165173 | 97796-102666 |
| Swit64 | switchgrass | 4980 | Ty1-copia | AC243240 | 52754-57733 |
