# Supplementary material for: A Highly Conserved, Small LTR Retrotransposon that Preferentially Targets Genes in Grass Genomes
Source: PLoS One. 2012 Feb 16;7(2):e32010. doi: 10.1371/journal.pone.0032010 (PMC3281118; doi:10.1371/journal.pone.0032010)
Supplement: Table S7 — List of primers for qRT-PCR. (DOCX) [file pone.0032010.s011.docx]

| Gene | Forward primer (5’-3’) | Reverse primer (5’-3’) | Note |
| --- | --- | --- | --- |
| Os02g43900 | ACGTCGACCATGACGACTACAA | CCTGAACTTGGCGATGAACTC |  |
| Os02g43906 | TCCGAGGCCTTGTCAACCT | GGGCGCTGTGTTTTCCATT |  |
| Os03g39020 | TGGCTTCCTCGACGACAGA | GCACGCCAATCAGATCGAA | Up insertion |
| Os03g39020 | CCCTCCAACGAGCCATCTAC | TCCTGGAGCTCGGCGTAAT | Down insertion |
| Os09g28180 | GCGAGGTTTAGCTACCAGGAGAT | CGCCGGATCCCACCTT |  |
| Actin | CTGACGCCGAGGATATCCA | GCCTTGACCATACCAGTTCCA |  |
